# Edible dormice (*Glis glis*) avoid areas with a high density of their preferred food plant - the European beech

**DOI:** 10.1186/s12983-017-0206-0

**Published:** 2017-04-20

**Authors:** Jessica S. Cornils, Franz Hoelzl, Birgit Rotter, Claudia Bieber, Thomas Ruf

**Affiliations:** Department of Integrative Biology and Evolution, University of Veterinary Medicine, Savoyenstraße 1, 1160 Vienna, Austria

**Keywords:** Habitat preference, ENFA, Foraging, Rodent, Pulsed resources

## Abstract

**Background:**

Numerous species, especially among rodents, are strongly affected by the availability of pulsed resources. The intermittent production of large seed crops in northern hemisphere tree species (e.g., beech *Fagus spec.*,oak *Quercus spec.*, pine trees *Pinus spec.*) are prime examples of these resource pulses. Adult edible dormice are highly dependent on high energy seeds to maximize their reproductive output. For juvenile dormice the energy rich food is important to grow and fatten in a very short time period prior to hibernation. While these erratic, often large-scale synchronized mast events provide overabundant seed availability, a total lack of seed production can be observed in so-called mast failure years. We hypothesized that dormice either switch territories between mast and non-mast years, to maximize energy availability or select habitats in which alternative food sources are also available (e.g., fleshy fruits, cones). To analyze the habitat preferences of edible dormice we performed environmental niche factor analyses (ENFA) for 9 years of capture-recapture data.

**Results:**

As expected, the animals mainly used areas with high canopy closure and vertical stratification, probably to avoid predation. Surprisingly, we found that dormice avoided areas with high beech tree density, but in contrast preferred areas with a relatively high proportion of coniferous trees. Conifer cones and leaves can be an alternative food source for edible dormice and are less variable in availability.

**Conclusion:**

Therefore, we conclude that edible dormice try to avoid areas with large fluctuations in food availability to be able to survive years without mast in their territory.

## Background

Pulsed resources, i.e., large-magnitude, low frequency, and short duration events of increased resource availability, have a huge effect on life-history traits of an individual (e.g., survival and reproduction) as well as on the dynamics of, populations and even whole ecosystems [1–3]. The intermittent production of large seed crops in northern hemisphere tree species (e.g., beech *Fagus spec.*,oak *Quercus spec.*, pine trees *Pinus spec.*) are prime examples of resource pulses. While these erratic, often large-scale synchronized mast events provide overabundant seed availability, a total lack of seed production can be observed in so-called mast failure years. Since these tree species show unpredictable masting patterns and are unable to yield seeds in two consecutive years, the differences in food availability are extreme, changing from overabundant to completely absent especially in years following a full mast event [4, 5]. The phenomenon of mast synchrony can be explained by three widely tested and supported mechanisms. Firstly, trees may swamp seed predators with as many seeds as possible to enhance the chances of seedling survival (the predator satiation hypothesis, [6–9]). Secondly, seed predators that are swamped in mast years may actually cache more seeds than they are able to retrieve, which would benefit seed dispersal and germination [10]. The third explanation involves weather conditions, which may either enhance or impair pollination success directly, or may affect flowering because selection has favored plants that all respond to weather characteristics in the same way, resulting in high synchrony [11]. Under all these scenarios synchrony between individual plants maybe further enhanced by long-term effects of the depletion of resources in masting years.

Numerous species, especially among rodents, are strongly affected by the availability of pulsed resources. In mice (e.g., *Apodemus flavicollis, Apodemus sylvaticus, Peromyscus leucopus, Peromyscus maniculatus*) and the bank vole (*Myodes glareolus)*, for example, mast events of beech and oak can cause a rapid population growth and an increased overwinter survival, while abundances are declining when the resource is depleting (e.g., [12–16]).

The impact of resource pulses on reproduction, survival, or hibernation patterns could be shown in several studies (e.g., [13, 17–20]). Eastern chipmunks (*Tamias striatus*) feast on seeds in autumn and store more nuts over the winter in mast years. Thus, this species can manage to raise two litters, with even higher juvenile survival in years with seed masting [21]. Interestingly, tree squirrels (both *Tamiasciurus hudsonicus* and *Sciurus vulgaris*) as well as the arboreal edible dormouse (*Glis glis*) are capable to anticipate future mast events; all three species increase their reproductive investment prior to the actual mast [17, 18, 22]. However, our knowledge on how the habitat choice in a species adapted to pulsed resources and is affected by strong fluctuations in food availability is very limited (but see [13, 23]). For consumers of seeds that show strong year-to-year variation in abundance, at least two different scenarios seem possible: (1) Switching territories between mast years and non-mast years. This “mast-tracking” option should be mostly available to species that are capable of travelling large distances, such as large mammals or birds [24, 25]. (2) Finding a habitat that, in addition to fluctuating seed resources, also provides alternative food sources (e.g., fleshy fruits, cones) in non-mast years. These alternative food sources should be most important for small, non-volant mammals, such as rodents. Year-to-year changes in the composition of food resources will be most relevant, however, for species that are long-lived enough to actually experience both mast seeding and mast failure years. Further, long-term effects of habitat characteristics evidently require a certain degree of site fidelity.

Among the seed-predating rodents, one species that appears to fulfill both of these criteria is the edible dormouse (*Glis glis*). Despite their small size (~100 g), these arboreal hibernators have a maximum longevity of 13 years [26], and hence may be exposed to varying masting situations. Further, previous studies have pointed to a high site-fidelity in edible dormice [18, 23, 27]. Especially for adult females it is known that they only travel as far as necessary from their nesting site to find suitable food [28].

In edible dormice mating occurs after hibernation only in mast years and juveniles are born very late in the active season (end of July to August; only one litter per year in central Europe), just in time with the ripening of beech seeds. Energy rich seeds are crucial for juveniles to grow and gain sufficient body fat stores before their first hibernation season [18, 23, 29]. Since these high caloric seeds are so important for juvenile survival, the optimal habitat for a reproductive female should include masting beech trees to maximize energy availability already during lactation. On the other hand, dormice have to cope in these habitats with low seed availabilities in mast failure years. One adaptation to this extremely reduced food availability is that they entirely skip reproduction in years with mast failures [18, 29]. Interestingly, however, survival rates in adult dormice are even higher in mast failure years than in reproductive years [18, 19, 23, 29]. While this is partly explained by extremely long hibernation seasons, during which the animals escape from predation by remaining hidden in their underground hibernacula [20, 23, 30], not all individuals can afford this strategy. Especially dormice with low body fat reserves have to stay active in mast failure years [20]. Indeed, the daily amount of time spent foraging did not differ in fall between a mast year and a mast failure year (Bieber et al. submitted).

For our analysis we used extreme situations, either non-mast or mast failure years with almost no trees producing seed buds/seeds at the whole study site, or full mast years, with almost all trees flowering. We used pollen densities as an indicator, but since pollen densities may not reflect seed densities in all species (e.g. [11]), we additionally confirmed seed densities visually in the field. However, edible dormice already have to know in spring whether a year will be a full or non-mast year, because growth of gonads and mating has to occur right away, to make sure ripe beech seeds are available to juveniles for prehibernation fattening [31]. Therefore the vast majority (~90%) of the adult dormice establish their territory already before July 15th (unpublished data), which is another indicator that they estimate the amount of future mast by assessing either pollen densities or seed bud densities [32]. Since pollen densities are highly correlated with the number of juveniles born per year (r = 0.97), both of these mechanisms are possible [33].

Adult edible dormice can gain weight relying on alternatives like leaves or fleshy fruits [23, 34]. A population of edible dormice in Crete has even been shown to survive in *Pinus brutia* forests, but it is not clear if these conifers are their only food source [35]. However, deciduous forests containing beech trees are the preferred habitat resulting in a higher lifetime reproductive success [36].

To date, there has been no systematic investigation of either site-fidelity or of the variables that determine habitat choice in this species. Therefore, we used data from a 9-year capture-recapture study of dormice encountered in a ~15 km^2^ area in the Vienna woods to first determine movements of juveniles, yearling, and adults between nest-boxes. Data on nest-box occupation by a total of ~1100 dormice were also used to see whether all potential territories were used equally, or if certain nest-box locations were preferred. Finally, to assess which type of habitats, if any, are favored and to determine variables that define habitat suitability, we computed an environmental niche factor analysis (ENFA; [37]). This method compares the available niche in a defined space with the area the species is actually using [37, 38]. For this analysis we used capture–recapture data and determined which environmental factor (assessed via a forest inventory of the areas surrounding each nest-box) affected the distribution of individuals at our study site.

## Methods

The study site was located close to St. Corona in the Vienna Woods (Lower Austria, 48°05'N/15°54'E; 400–600 m asl). The area (size ~15 km^2^) is covered by a mixed forest with most of the site dominated by deciduous beech forest (~60% of the trees) and ~30% coniferous trees.

There were 124 wooden nest-boxes (fixed at 2–3 m height on trees) randomly distributed along the forest trails, which were checked for the presence of edible dormice every second week (May-October; 2006-2014). In the active season, edible dormice use these nest-boxes (in place of natural tree holes in primeval forests) to rest during the day and raise their young. Every newly captured dormouse was marked with a subcutaneously injected PIT-Tag transponder (BackHome BioTec®, 13.8 mm × 2.1 mm or Tierchip Dasmann®, 12.0 mm × 2.0 mm). All dormice were sexed and classified as either juvenile (J, before the first hibernation), yearling (Y, before the second hibernation period) or adult (A, after the second hibernation period) using fur color and size given in Schlund [39].

We recorded environmental variables using wide-ranging forestry based GIS data in a 100 m radius around each nest-box from 2006 with ArcGIS 9.1 ([40]; geographic information system; Table 1). This use of a 100 m radius was based on home ranges determined in three telemetry studies in populations with different densities of edible dormice [28, 41, 42]. Nest-box locations were obtained using a 12-channel GPS receiver (eTrex® Summit, GARMIN Corporation). Small-scale parameters were documented for a 30 m radius around each nest-box (Table 1). Tree species with a very low coverage of the area (mean under 2%) were excluded from the analysis. Herb cover was also excluded from the analysis, because the animals rarely dwell on the ground [23]. A total of seven variables were included in the model to explain the distribution of edible dormice (Table 1).

**Table 1:** Environmental variables determined either from a GIS based forest inventory or small scale measurements in the forest

Source	Variable	Abbreviation	Unit
GIS based	Altitude	“alt”	m above sea level
100 m Radius	Forest age	“age”	years
	*Fagus sylvatica* density	“fag”	%
	Conifer density	“conifers”	%
	Slope	”slope”	degrees
Small scale	Canopy closure	“can”	25%, 50%, 75%, 100%
30 m Radius	Girth of nest-box tree at breast height	“girth”	cm

Canopy closure was measured by partitioning the space around the crown into four quadrants and considering the connection between this crown and neighboring trees. The variable is equal to 100%, if all four quadrants are connected

The density of beech and coniferous trees were included because their seeds and leaves are important food items of edible dormice [34]. Forest age, canopy closure, and slope of the hillside were used because these variables affect the structure and stratification of the forest, which may affect both foraging opportunities and predation risk of the animals. We also included the girths of the trees (as a proxy for both age and height) at which the nest-boxes were mounted on, because dormice may select habitats based on the suitability of the immediate nesting site. Further, we included altitude of the location, as even small differences in elevation can have an influence on the microclimate of the forest.

### Statistics

#### Movements between areas

To analyze dormouse movements between nest-boxes, we calculated distances travelled as well as the time between capture and recapture, the mean and maximum number of captures, mean total distance and the number of nest-boxes used. Animals that had not been recaptured were excluded from the analyses.

To investigate to what extent edible dormice move between nest-boxes in the different age classes, we computed a linear mixed effects model [43] to analyze the mean distance the animals covered using sex, age and timespan between captures as explanatory variables. To adjust for repeated measurements the individual ID was used as a random effect. The response variable distance was log-transformed to achieve a normal distribution of the model residuals, which was confirmed with a Shapiro-Wilks test. To include mast in our analysis we also calculated a linear mixed effects model [43] containing sex, mast and timespan. We could not include age here, because the category juveniles and yearling, were directly associated with full and non-mast year, respectively. We also included individual ID as a random factor in this model and log transformed the response variable mean distance. Subsequently we calculated a type 3 anova in both models. We used a chi^2^ test to test if the nest-box occupation was equally distributed over the whole study site.

#### Environmental niche factor analysis

The environmental preferences of the edible dormice at our study area were calculated using the ENFA approach. The number of captures per nest-box was used as the response variable. This model is based on the concept of the ecological niche and compares the available niche to the niche the species is using. The advantage of this analysis, compared to classical methods, is that it is solely based on presence data. A principal component analysis (PCA) as the first step ensures that the appropriate weights and transformations are provided for the subsequent ENFA. The ENFA summarizes variables into a few uncorrelated factors (as does the PCA), but the so- called marginality and specialization axes have ecological meaning (for details see [38]). We made sure that the environmental variables we included in the ENFA were not highly correlated (all r < 0.5).

In ENFA, the marginality axis is the direction on which the species niche differs at most from the available conditions. After removal of the marginality, a specialization factor can be determined by computing the direction that maximizes the ratio of the variance of the global distribution to that of the species distribution. This determination of the specialization axes is repeated until all the information has been explained [44]. A large specialization corresponds to a narrow niche relative to the habitat conditions available to the species.

For the illustration of the ecological niche in a defined space, we used biplots with one marginality axis and the first specialization axis [38, 44]. The environmental variables were plotted as arrows, where the length of the arrow is a measure of the influence of the variable on the position of the niche in the available habitat. That means the longer an arrow, the more important it is for the explanation of the marginality axis [44]. For the coefficients of marginality a positive value means that the species prefers values higher than the mean, while a negative coefficient indicates a preference for lower values with respect to the study area [37]. The specialization factors have to be handled as absolute values, they represent the variance ratio of the variables. The higher this factor, the higher the degree of specialization with respect to the variable, signs are arbitrary [37]. Since the first specialization axis explains most of the variance (also seen in the histograms in the upper right corner of every ENFA analysis; Figs. 3 and 4) we only used this first axis for the individual biplots. To test if our defined marginality and specialization axes were significant we performed a Monte-Carlo randomization test with 1000 permutations. All analyses were carried out using R version 3.1.1 [45] with the package ‘adehabitatHS’ [46]. We calculated the overall situation for all years and conditions (Fig. 3), performed separate ENFA’s for female and male dormice (Fig. 4a + b), and additionally for the two extreme food situations in two non-mast and two full mast years (Fig. 4c + d).

#### Simulations

To investigate the effect of the presence of multiple tree species on the overall year-to-year variability in seed availability, we simulated time series with a given coefficient of variation using the R function rnorm. To test the effect of different degrees of synchrony in seed production between tree species on mean temporal variability, we simulated correlated time series (length 200 years, CV 0.5), with correlation coefficients randomly varying around given desired r-values, ranging from 0 to 1 (Fig. 5b). Each point was determined as the mean from 1000 repeats.

## Results

### Nest-box occupation and movements

**Fig. 1:**
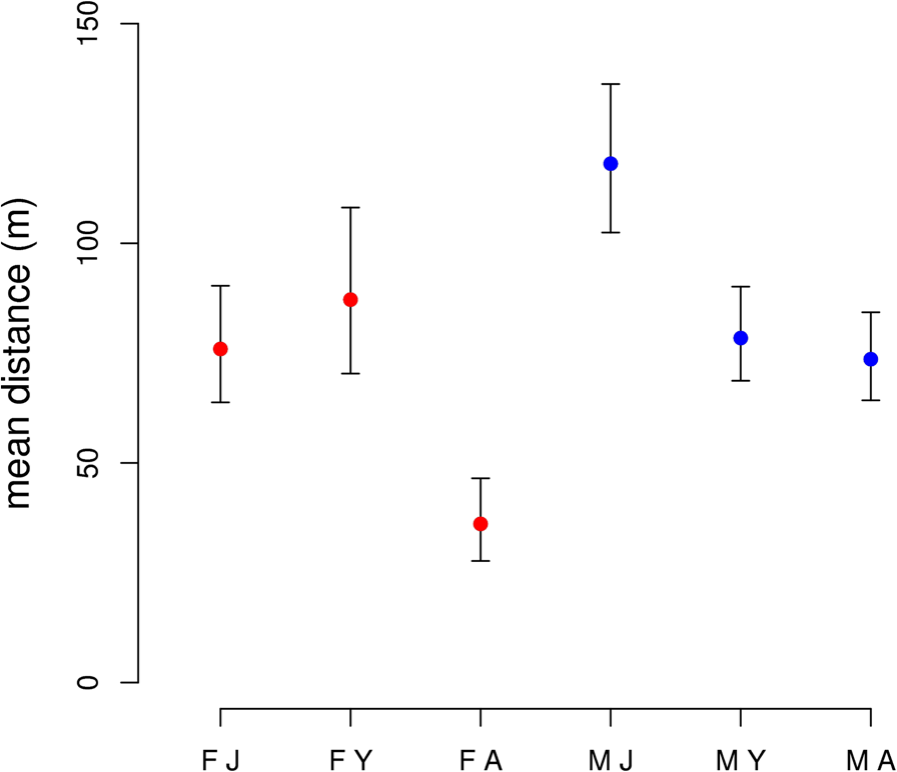
Mean distance ± SEM of relocations in the different age classes of edible dormice (FJ = juvenile female; FY = yearling female; FA = adult female; MJ = juvenile male; MY = yearling male; MA = adult male)

From 2006 to 2014 we caught 1189 individual dormice at our study site. Overall there were 5950 capture events and the mean number of nest-boxes used per animal was 1.58 ± 0.02. One animal was captured 29 times over the course of nine years; the mean number of captures was 3.26 ± 0.06. Overall, a high proportion of animals were captured in the same (52.7%) or neighboring nest-boxes over the years, with especially adult females showing high site fidelity, by staying in the same box. Juvenile dormice and particularly juvenile males had a higher tendency for dispersal and moving longer distances into other areas of the study site, or presumably also into the adjacent forest areas outside of our study area (sex: F = 16.11, *P* < 0.001; age: F = 36.01, *P* < 0.001, sex:age: F = 19.33, *P* < 0.001, timespan: F = 32.57, *P* < 0.001; Fig. 1).

The mean distance travelled was 49.44 ± 2.62 m for all age and sex categories, also indicating high overall site fidelity. In the second model, were we included mast, sex still had a significant influence (sex: F = 7.71, *P <* 0.007), but there was no significant result for timespan (F = 2.69, *P* = 0.134), but a significant difference between non-mast and mast years (F = 12.15, *P <* 0.005), with adult animals travelling mean distances of 87.7 ± 73.02 m in full mast years and 125.4 ± 91.6 m in non-mast years.

**Fig. 2:**
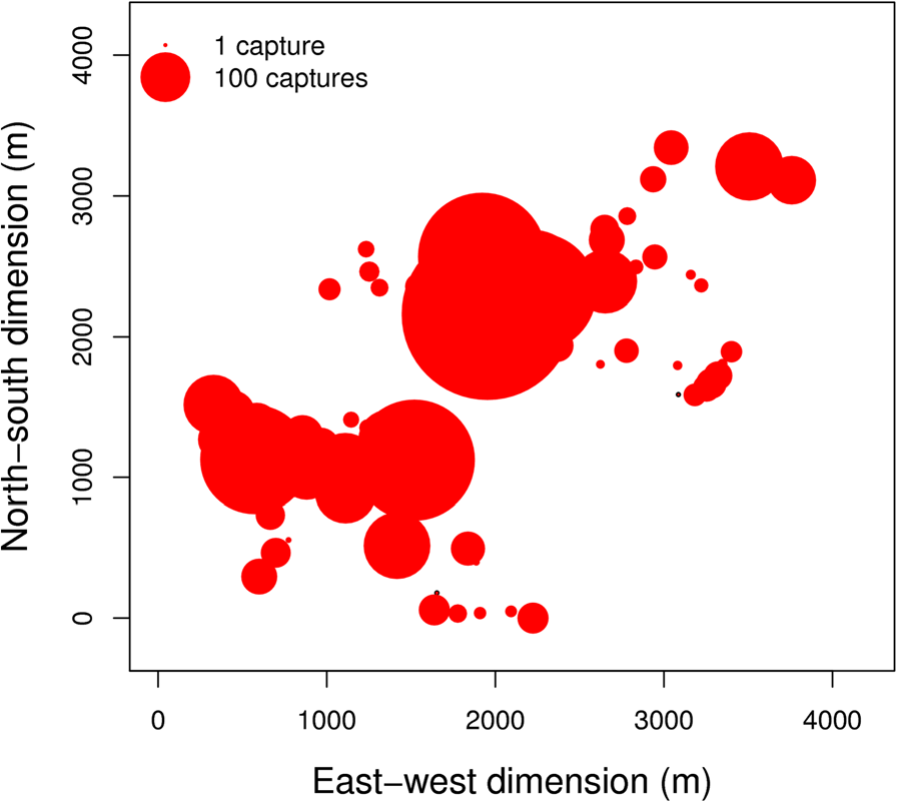
Distribution of captured animals in the nest-boxes from 2006 to 2014. The sizes of the dots reflect the number of captures

Nest-box occupation was unequally distributed over the study site (χ^2^ = 632.26, df = 119, *P* < 0.001). There were sections of the study site in which nest-boxes were more frequented than in other parts, and some of the nest-boxes were only used rarely during the time of the study (Fig. 2).

### Environmental influences

**Fig. 3:**
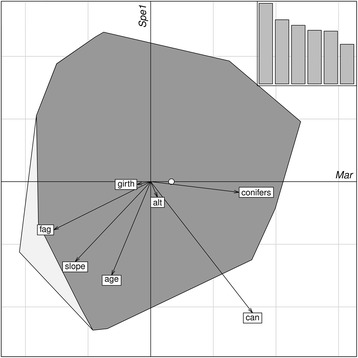
Result of the overall ENFA carried out to determine the relationship between environmental variables and the distribution of edible dormice in the study area (years 2006–2014). The eigenvalue diagram of the analysis in the *upper right* corner shows the contribution of each specialization axis to the overall specialization, were each barplot represents one specialization axis (*Spe* 1–6; only). The biplot for the analysis is formed by the marginality (x-Axis; *Mar*) and the first of these specialization axes (y-Axis, *Spe1*), which explains most of the variance. The *light grey* area represents the minimum convex polygon enclosing all the projections of the available habitat, whereas the *dark grey* area corresponds to the habitat used by the animals. The *white dot* represents the centroid of the used habitat, while the origin of the plot is the centroid of the available sites. The environmental variables are projected via the *arrows*. The longer an *arrow*, the more important it is for the explanation of the marginality axis. The arrows that have the biggest angle from the marginality axis have the highest specialization, signs are arbitrary in this case. Environmental variable abbreviations: alt = Altitude; age = Forest age; fag = *Fagus sylvatica* density; conifers = Conifer density; slope = Slope; can = Canopy closure; girth = Girth of the nest-box tree. For further explanations of the variables see Table 1

**Fig. 4:**
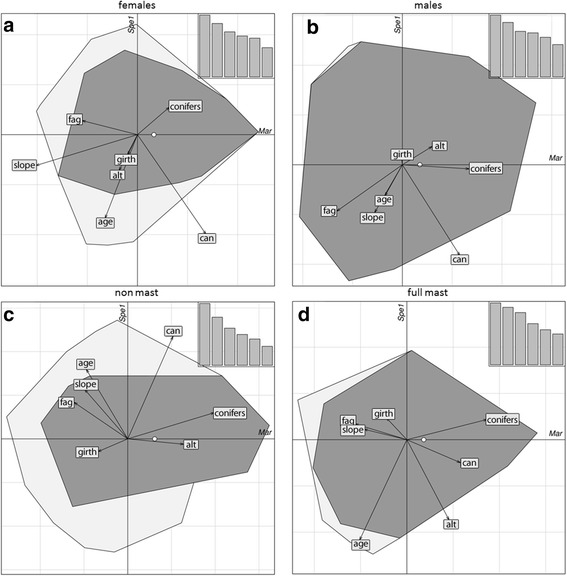
Results of the ENFA’s carried out to determine the relationship between environmental variables and the distribution of edible dormice in **a** two non-mast years 2012 + 2014 **b** two full-mast years 2011 + 2013 **c** only females 2006–2014 and **d** only males 2006–2014. For the detailed explanation of the graphical parameters see Fig. 3

To explain this unequal distribution of animals throughout the forest and to assess which factors influenced their site fidelity we performed different ENFA’s. These analyses showed that the eigenvalues of the specialization decreased after the first axis for all of the tests performed. Hence, the first axis explained most of the specialization, allowing us to use the first specialization axis only, in all of the computed ENFA’s (Figs. 3 and 4).

However, the deviation between available and used niche was moderate in all of the analyses (distance of the available niche to the centroid: overall = 0.1097, non-mast = 0.3801, full-mast = 0.1333, females = 0.1077, males = 0.1234; see also white dots (x-Axis in Figs. 3 and 4)). Especially for the overall analysis, for males and for full-mast years there was an almost complete overlap between used and available niches (Figs. 3 and 4b and d). When the analysis was restricted to non-mast years or to females only, the overlap was far less pronounced (Fig. 4a and c).

In the overall analysis, canopy closure (preferred), beech tree proportion (high densities avoided), conifer density (high density preferred) and slope of the terrain (large slopes avoided) were the most important variables that defined the used habitat. The highest specialization values in the overall analysis showed that edible dormice were restricted to a limited range of areas with high canopy closure, relatively young trees (at an age of seed production onset) and moderate slopes (i.e., a low tolerance of variation in these three variables, see Table 2 and Fig. 3). The specialization value for conifer density, however, was very low as illustrated by the short distance to the x-Axis in Fig. 3. In other words, the animals used a wide range of the available conifer cover.

**Table 2:** Coefficient values for all calculated ENFA models for the seven environmental variables included

	overall		Non-mast		Full-mast		Females		Males	
	Mar	Spe1	Mar	Spe1	Mar	Spe1	Mar	Spe1	Mar	Spe1
fag	**−0.52**	−0.26	**−0.38**	0.25	**−0.38**	0.12	**−0.39**	0.09	**−0.55**	−0.39
can	**0.54**	−0.70	0.32	0.72	**0.39**	−0.17	**0.48**	−0.69	**0.48**	−0.76
alt	0.04	−0.08	**0.39**	−0.03	0.31	−0.59	−0.13	−0.25	0.25	0.16
age	−0.21	−0.50	−0.29	0.49	−0.34	−0.74	−0.23	−0.58	−0.15	−0.26
girth	−0.07	−0.02	−0.21	−0.09	−0.15	0.16	−0.07	−0.14	−0.01	0.05
slope	−0.40	−0.43	−0.3	0.34	−0.32	0.07	**−0.71**	−0.21	−0.23	−0.40
conifers	**0.47**	−0.06	**0.6**	0.18	**0.59**	0.14	0.22	0.19	**0.56**	−0.03

The three highest values concerning the marginality are printed bold for all models. Abbreviations same as for Table 1

Males avoided areas with high densities of beech trees more than females (Fig. 4 a + b). In addition males preferred areas with high density of coniferous trees, whereas in females the preference for high conifer density was less pronounced. The specialization of females on forest stands of lower age was more prominent than among males. This was in contrast to the slope of the terrain, where males showed a higher specialization on moderate slopes. Both sexes had a high preference for closed canopy (Fig. 4a + b, Table 2).

There was no major shift in habitat use between full- mast and non-mast years. Despite the difference in food availability in those types of years, dormice remained in habitats with a relatively large density of conifers and a lower density of beech trees (Fig. 4c + d).

## Discussion

### Site fidelity

Our long-term analysis indicates that edible dormice, especially adult females, show high site fidelity and often stay at the same site over several seasons (Fig. 1). Adult males also showed high overall site fidelity (Fig. 1) and only moved over slightly longer distances in non-mast years, probably due to lower above-ground abundance and competition for good territories in those years (see below). As is typical for rodents in general [47], the only group with an above-average tendency for dispersal was juvenile males, which covered longer distances to explore new territories (Fig. 1). They face a trade-off in the year of their birth between investing in fattening in the mothers territory or exploring new habitats. Dispersal may improve the chances of reproduction for juvenile males in the subsequent year, but may also lead to a higher risk of predation in a foreign territory or during dispersal [47–49]. Our data on the high overall site fidelity of adult dormice confirm previous, shorter studies on this question [18, 27, 35] and further demonstrate that our environmental analysis around the nest-boxes most likely covered the majority of the animals’ home ranges.

### Habitat choice

**Fig. 5:**
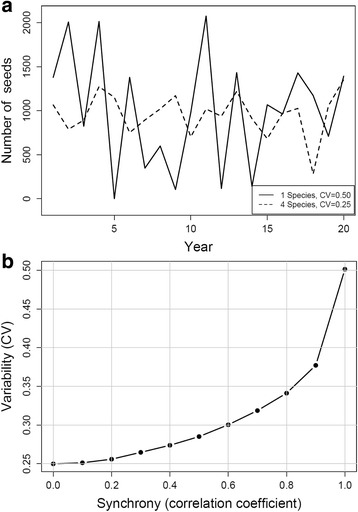
**a** Simulated seeding variability over a 20 year period. The *solid line* shows seeding fluctuations in a single tree species (e.g. beech) showing a coefficient of variation (CV) of 0.5. The *dashed line* shows the collective (mean) fluctuation of 4 tree species, e.g. conifers, which individually show the same CV (0.5) and vary independently. The collective CV of 4 species is reduced by 50% (CV = 0.25). **b** Simulated effect of different degrees of synchrony between 4 tree species on mean seed variability. All species show individually the same CV (0.5). Note that even at a correlation coefficient of 0.5, the mean CV (0.28) is strongly reduced below 0.5

The ENFA analysis showed a large general overlap of the used and available niche space (Figs. 3 and 4), indicating that most of the areas around the nest-boxes were a suitable habitat for edible dormice. More importantly, our results indicate that sub-areas of our study site were sufficiently heterogeneous to allow us to identify several habitat characteristics that are clearly preferred or avoided. The most surprising result of our environmental niche analysis was that edible dormice, despite their dependency on beechnut availability for reproduction, avoid forest stands with high beech density and prefer areas with a large proportion of coniferous trees. It has long been known that conifer cones and leaves can be an alternative food source, but there was never an indication for a preference of conifer forest stands [34, 50]. Coniferous trees also have fluctuating masting events, and coefficients of variation (CV) in seed production in individual conifer species are not smaller than in beech (review in [6]). However, in most forests in the distribution range of edible dormice there is only one beech species (*Fagus sylvatica*) but often there are several species of conifers. It can be shown that, when the number of conifer species reaches 4, as was the case at our study site, their collective coefficient of variation in seed production is only half that of a single tree species, even if all species have the same CV individually (Fig. 5). Since edible dormice can forage on different species of conifers, this results in conifers being a more stable food source. This effect will be reduced if seed production among tree species varies synchronously among conifers, but this degree of synchrony seems only moderate (r < =0.5; [51]). At low to moderate levels of synchrony between tree species, the effect of the presence of multiple species on reducing see variability is still strong (Fig. 5b). Accordingly, Ruf et al. [18] found that dormice in mixed beech and conifer forests survived even better after years of reproduction skipping than dormice in forests dominated by beech. Hence, it seems the optimal habitat for dormice are forest stands that provide a fairly steady food resource such as various conifer seeds, interspersed with a relatively low proportion of trees that show variable masting but produce large seeds, namely beechnuts or acorn, with high energy content [34].

Edible dormice have an alternative option to either stay in their territory and live with lower food availability or move to another site. They can respond to the absence of beech seed not only by foregoing reproduction in certain years, but can also estivate throughout summer, without any food intake [20]. In dormice, states of dormancy in underground burrows are associated with extremely high survival rates, which are thought to reflect low predation risk [19, 20, 52, 53]. Indeed, while remaining largely motionless and odorless in closed hibernacula located ~50 cm below the surface [36], dormice are protected from most predators, particularly from owls, their main predators [35]. Predator avoidance is also a central factor in the high longevity of *Glis glis*, with free-living animals reaching an age of up to 13 years [26, 30].

Because reducing the risk of external mortality is an essential characteristic of the life-history of edible dormice, we suggest that several of the other habitat preferences identified here are also related to minimizing predation risk. The amount of time terrestrial animals spend foraging is mainly influenced by the availability of habitat structures that lower predation risk [54]. Not surprisingly then, one of the most suitable habitats identified by the overall ENFA seem to be closed canopy forests with 75 to 100% closure. Closed canopies should be advantageous in terms of predation avoidance and foraging, as they hamper attacks by birds of prey from above, and allow the animals to move easily between adjacent trees. Further, large birds of prey like owls with wingspans of up to 250 cm (*Strix aluco*) cannot maneuver quickly in forest areas with dense vegetation and therefore prefer open, mature forests as their major hunting grounds [55, 56]. This factor would explain why dormice prefer younger over old-growth forests, as younger stands show higher vertical stratification and mid-canopies [57], which are avoided by owls [55]. Finally, predator avoidance could also explain why dormice avoid stands on steep slopes, because slopes cause layering and vertical opening of the canopy, which may lead to increasing hunting opportunities for birds of prey, especially because owls hunt better under illuminated conditions [58–60].

### Males vs females

Females seem to use a slightly smaller proportion of the available habitat (convex polygons; Fig. 4a + b). This higher degree of specialization among females may well be related to their higher costs of reproduction, which requires optimal food resources and foraging conditions. There was a tendency of males to prefer conifer stands and avoid areas with high beech tree proportions even more than females (Table 1). This can be explained by the fact that females share territories with their juveniles, which are highly dependent on energy-rich beechnuts to gain sufficient weight before hibernation [18, 29]. Females with offspring also show a heightened aggressiveness and vigorously defend nest-boxes against intruders in mast years with reproduction (J.S.C. unpublished observation). This observation is in line with the high site-fidelity of adult females (Fig. 1). If females defend their nesting sites this should lead to reduced shelter availability for males (and non-reproductive females) after mating, until weaning of the juveniles. Consequently, males may be forced into territories with higher conifer density, which also provide a good canopy closure, and may have to alter their foraging behavior. This diversification of foraging preferences with different amounts of beech availability was also found by Schlund et al. [31], who detected similar densities of edible dormice in a beech forest with 70% and a coniferous mixed forest with 20% beech trees. The rate of juveniles per female however was far lower in the coniferous-mixed forest than in the beech forest [31], matching our finding of less avoidance of beech among adult females.

### Non-mast vs full-mast

In non-mast years the occupied niche was a smaller fraction of the available niche than in full-mast years (Fig. 4c + d), indicating that dormice were apparently more selective in years of mast failure. We attribute this observation to the fact that in non-mast (non-reproductive) years the number of dormice occupying nest-boxes during the active season is ~50% smaller than in mast/reproductive years [19, 20, 26]. This is because in non-mast years a large fraction of the animals, in particular those individuals that have high body fat reserves in spring, retreat to underground burrows for estivation [20]. Hence the abundance of dormice above-ground will significantly differ between years [19, 20]. This provides those animals that remain active and foraging with the opportunity to choose among a larger number of unoccupied nest-boxes in good habitats, which is also reflected by movements over larger distances in non-mast years.

There was, however, no noticeable change in the preference or avoidance of specific habitat characteristics between mast and non-mast years (Table 1), despite lower abundances and a higher mean distance travelled. Together with the overall high site fidelity this finding suggests that, rather than switching between territories with different characteristics, dormice tend to occupy and remain in areas which provide optimal long-term conditions, and which buffer short-term fluctuations in mast seeding.

## Conclusions

We showed for the first time that edible dormice avoid forest stands with a high density of beech, likely to evade exposure to large fluctuations in food resources caused by extremely pulsed beech seeding, following the theory of risk sensitive foraging (i.e., risk averse; [61]). This behavior is still fully compatible with the fact that dormice require energy-rich seeds for successful reproduction. It has been estimated that the amount of seeds produced by beech or oak in a full-mast year is ample enough to allow all granivores in a deciduous forest to live ad libitum on beechnuts or acorn alone [62]. Accordingly, a single beech tree in a dormice territory is almost certainly sufficient to provide a female and its offspring with adequate food resources for growth and prehibernation fattening [63]. Interestingly, most other habitat preferences of dormice, such as closed canopies and younger stands with vertical stratification appear to be related to minimizing predation risk, which is a main reason for animals to switch foraging grounds [54]. This points to a potential tradeoff between optimizing resource allocation and predator avoidance, which would be expected from the optimal foraging theory, (e.g. [58, 64, 65]), but deserves further investigation in this species.
